# Diagnostic Value of Multimodal Imaging and Histopathology in Gallstone Ileus: A Retrospective Analysis

**DOI:** 10.3390/diagnostics15162017

**Published:** 2025-08-12

**Authors:** Alina Cristiana Venter, Ovidiu Țica, Andrada Cheseli, Cristian Marius Daina, Ioan George Oswald, Corina Beiușanu, Ginetta Andreescu, Ilarie Brihan

**Affiliations:** 1 Department of Morphological Disciplines, Faculty of Medicine and Pharmacy, University of Oradea, 410073 Oradea, Romania; aventer@uoradea.ro (A.C.V.); cbeiusanu@uoradea.ro (C.B.); gandreescu@uoradea.ro (G.A.); 2 Radiology Clinic, Emergency County Clinical Hospital of Bihor, 410165 Oradea, Romania; anda.cheseli@gmail.com (A.C.); ioanoswald@uoradea.ro (I.G.O.); 3 Department of Pathology, Emergency County Clinical Hospital of Bihor, 410165 Oradea, Romania; 4 Department of Surgical Disciplines, Faculty of Medicine and Pharmacy, University of Oradea, 410073 Oradea, Romania; cdaina@uoradea.ro; 5 Clinic of Anaesthesiology and Intensive Care, Emergency County Clinical Hospital of Bihor, 410165 Oradea, Romania; 6 Department of Preclinical Disciplines, Faculty of Medicine and Pharmacy, University of Oradea, 410073 Oradea, Romania; 7 Department of Psycho-Neurosciences, and Recovery, Faculty of Medicine and Pharmacy, University of Oradea, 410073 Oradea, Romania; ibrihan@uoradea.ro

**Keywords:** gallstone ileus, biliary-enteric fistula, computed tomography (CT), histopathology, multimodality

## Abstract

**Background:** Gallstone ileus is a rare cause of bowel obstruction, often presenting with nonspecific symptoms that delay diagnosis. This study assessed the diagnostic value of CT imaging and its correlation with histopathological findings in confirmed cases. **Methods:** A retrospective analysis of 14 patients with surgically confirmed gallstone ileus was performed. All underwent abdominal radiography, ultrasound, and CT. Imaging findings were evaluated for calcification type, impaction site, and fistula presence. Histopathology from surgical specimens was used for correlation. Relative risk (RR) and 95% confidence intervals (CIs) were calculated, with adjustments for small sample size. **Results:** Rim-calcified gallstones were the most common (50%) and significantly associated with chronic inflammation (RR 1.42, 95% CI: 1.05–1.93). Cholecystoduodenal fistulas were most frequent (57.1%), with a 92.8% imaging-pathology concordance. Ileal impaction (35.7%) was linked to increased risk of bowel necrosis (RR 2.0, 95% CI: 1.1–3.6). All stones were >3 cm; two patients had recurrence. No perioperative mortality occurred. **Conclusions:** CT imaging demonstrates high diagnostic accuracy and strong correlation with histopathological findings in gallstone ileus. Identifying calcification patterns and impaction sites aids early diagnosis and surgical planning.

## 1. Introduction

Gallstone ileus is a rare and potentially life-threatening cause of mechanical bowel obstruction, typically occurring in elderly individuals with a history of chronic gallbladder disease. Although it represents only 1–4% of all cases of mechanical small bowel obstruction, its clinical significance lies in the diagnostic challenges and elevated perioperative risk associated with delayed intervention [1,2,3]. Women over the age of 70 are particularly susceptible, reflecting both hormonal influences on bile composition and age-related gallbladder dysmotility [4]. Chronic gallbladder inflammation may lead to fistula formation—most commonly cholecystoduodenal—through which large gallstones pass into the bowel and become impacted, typically in the terminal ileum [1,5]. Among patients with colonic gallstone ileus, the sigmoid colon was identified as the most common site of impaction, consistent with the published literature [6]. However, if the gallstone progresses into the colon, the sigmoid colon—being the narrowest segment—is the most frequent site of impaction in colonic gallstone ileus, as highlighted by Augustin et al. [6]. In cases of colonic gallstones, particularly with sigmoid impaction, surgical removal is no longer considered the first-line intervention. Instead, colonoscopic removal using extraction snares, lithotripsy, or mechanical fragmentation is increasingly used as an initial, less invasive treatment approach. Chronic cholecystitis can cause erosion of the gallbladder wall into adjacent bowel, leading to fistula formation—most commonly cholecystoduodenal—which enables gallstone migration into the intestinal tract [7]. The variability in fistula formation and gallstone size contributes to the diverse clinical and radiological manifestations [8].

The clinical presentation is frequently insidious and intermittent, with patients reporting vague abdominal pain, nausea, vomiting, distension, and constipation—the so-called “tumbling” phenomenon. This variable presentation often results in delayed diagnosis and increased morbidity, particularly among elderly individuals with comorbidities [1,2].

The classic presentation includes Rigler’s triad—pneumobilia, ectopic gallstone, and signs of bowel obstruction—though this is seen in less than 50% of cases on plain radiographs [9]. Computed tomography (CT) is now considered the diagnostic gold standard due to its high sensitivity and specificity, providing visualization of fistulous communication, gallstone characteristics, and associated complications such as ischemia or necrosis.

Imaging is critical for diagnosis. While abdominal radiography detects Rigler’s triad in only 14–30% of patients, computed tomography offers superior diagnostic yield—with sensitivity approaching 90–93%—and accurately demonstrates ectopic gallstone, pneumobilia, and obstruction [5,10]. Ultrasound may reveal pneumobilia or ectopic stones in some cases, being generally adjunctive [5]. Advances in imaging modalities, particularly multidetector CT and MRCP, have significantly improved diagnostic accuracy [11]. However, the need for histopathological correlation remains crucial to confirm inflammatory status, identify chronic changes, and evaluate for possible neoplastic transformation in long-standing fistulas [12]. This multidisciplinary diagnostic approach is particularly important given the high recurrence rate—up to 8%—after isolated enterolithotomy [13].

Surgical intervention remains the cornerstone of management. Evidence from a U.S. national database indicates that isolated enterolithotomy (ELT) alone (stone extraction only) is performed in approximately 62% of cases and is associated with lower perioperative mortality (6.7%) versus ELT combined with fistula repair or bowel resection, which carries significantly higher risks (OR ~2.9) [2]. Operative trend analysis confirms that ELT alone offers better outcomes and fewer complications, especially when patient risk is high [2,3]. In cases of colonic gallstone ileus—particularly with sigmoid impaction—surgical removal is no longer considered the first-line intervention. Instead, colonoscopic removal using extraction snares, lithotripsy, or mechanical fragmentation is increasingly used as an initial, less invasive treatment approach.

Minimally invasive approaches—including laparoscopic and endoscopic ELT—have shown promise in small series, especially for colonic or duodenal obstructions, but broader application remains limited by technical challenges. Recurrence rates following isolated ELT may reach 5–8%, underscoring the need for vigilant follow-up. In colonic gallstone ileus, recent evidence suggests that colonoscopic extraction techniques, including snare capture, lithotripsy, and mechanical fragmentation, may be effective first-line options, particularly for sigmoid impaction. These techniques have demonstrated success in selected cases and are incorporated into recent management algorithms [6].

In summary, gallstone ileus demands high clinical suspicion in elderly patients presenting with bowel obstruction and a history of biliary disease. Prompt CT imaging and judicious selection of ELT—alone or staged with fistula repair—are essential to optimize outcomes for this vulnerable patient cohort.

This study aimed to characterize the imaging findings, particularly gallstone calcification patterns and fistula types, from a radiological and pathological perspective in patients with surgically confirmed gallstone ileus, to enhance diagnostic accuracy and surgical planning. This study builds upon existing literature by providing an integrated analysis of imaging findings and histopathological characteristics in surgically confirmed cases of gallstone ileus. We aim to elucidate patterns of gallstone calcification, anatomical variations in fistula formation, and their implications for clinical management. The goal is to improve preoperative diagnosis, guide surgical planning, and ultimately enhance patient outcomes. However, the small sample size limits clinical reproducibility, underscoring the need for larger multicenter studies.

## 2. Methods

### 2.1. Patient Selection

This single-center, retrospective cohort study included consecutive patients with surgically confirmed gallstone ileus at our institution between January 2018 and December 2024. Eligibility criteria included (1) definitive intraoperative diagnosis of gallstone ileus, (2) availability of complete preoperative imaging (radiography, ultrasound, and CT), and (3) availability of histopathological specimens. Exclusion criteria included incomplete imaging data, ambiguous operative findings, or missing pathology reports. All patients included were consecutive cases meeting stringent inclusion criteria to reduce selection bias. A retrospective approach was chosen to leverage existing surgical and imaging datasets, allowing comprehensive radiologic-pathologic correlation in this rare condition where prospective recruitment is limited. Although the small sample size limits power, exact logistic regression models and Cornfield-adjusted confidence intervals were employed to mitigate statistical bias inherent in small datasets.

### 2.2. Data Collection

Clinical, radiological, and pathological data were systematically extracted from electronic medical records. Patient demographics, clinical presentations, radiological findings (calcification patterns, gallstone impaction sites, and fistula types), surgical procedures, and outcomes were collected and documented in a standardized data extraction form. To ensure data security and patient confidentiality, all patient data were anonymized, and identifiable information was removed before analysis. Data accuracy and completeness were independently verified by two investigators.

### 2.3. Imaging Evaluation

All imaging studies were performed using a high-resolution multidetector CT scanner (64 slice or higher) following institutional protocols, ensuring consistent imaging acquisition parameters (slice thickness ≤3 mm, pitch ≤1, and standard abdominal windowing). Imaging data were independently reviewed by two senior radiologists (ACV and AC), blinded to clinical and surgical outcomes. Radiological assessment specifically focuses on gallstone calcification patterns, classified into dense (fully calcified), slight opacification, or rim calcification. Gallstone locations, radiographic signs of obstruction, and associated findings such as pneumobilia were meticulously documented. Discrepancies were resolved by consensus.

CT scans were performed using a GE Revolution CT scanner, both 64 slice or higher, with collimation thickness of 0.6 mm, reconstruction intervals of 1–2 mm, and intravenous contrast when renal function allowed. Oral contrast was not routinely used due to the urgency of the cases. CT images were evaluated in axial, coronal, and sagittal planes using abdominal window settings. Radiographic classification of gallstones included size (measured in maximum axial diameter), degree of calcification (dense, rim, or faint), and number. Fistulous tracts were evaluated based on wall discontinuity, air-fluid levels, and gallbladder collapse.

### 2.4. Pathological Examination

Surgically extracted gallstones were submitted for comprehensive pathological evaluation. Macroscopic assessment included measurements of stone diameter and evaluation of surface characteristics. For histopathological examination, tissue samples from the gallbladder, surrounding fistulous tracts, and adjacent bowel wall were formalin fixed, paraffin embedded, and stained with hematoxylin and eosin (H&E). Microscopic evaluation was conducted by two experienced pathologists to assess inflammatory changes, fibrosis, granulomatous reactions, and mucosal integrity. The correlation of imaging and histological findings provided insights into the chronicity and structural impact of fistulous communication. Radiological and pathological findings were cross-validated to ensure diagnostic concordance. All samples were processed using standardized protocols to minimize inter-sample variability and ensure uniformity in histological assessment.

Resected specimens were formalin fixed within 30 min of surgical removal, with gross inspection performed by a senior pathologist. Tissue blocks were sectioned at 4-micron thickness and stained with H&E. Additional stains such as Masson’s trichrome and PAS were utilized where fibrosis or epithelial integrity needed further clarification. Chronic inflammation was defined by the presence of lymphoplasmacytic infiltrate, mucosal ulceration, and granulation tissue.

### 2.5. Interobserver Reliability (Added)

To strengthen the internal validity of radiological assessment, interobserver variability was analyzed using Cohen’s kappa statistic across the two radiologists (ACV and AC). Interobserver agreement was assessed using Cohen’s kappa statistics across two independent radiologists. Imaging features—including calcification type, fistula presence, and impaction site—were scored independently. A kappa value > 0.8 was interpreted as excellent agreement. Discordant cases were resolved through a third consensus review. Discrepancies were reviewed in consensus meetings.

### 2.6. Statistical Analysis

Descriptive statistics summarized patient demographics, imaging, and pathological characteristics. Continuous data were reported as means ± standard deviation, and categorical variables as frequencies and percentages. Comparative analysis between categorical groups (calcification types and impaction locations) utilized Chi-square tests. Relative risks (RRs) were computed where appropriate. To address potential biases and minimize overfitting due to small sample sizes and event rates, statistical methods specifically suited for small datasets, including exact tests and adjusted confidence intervals, were employed. Beyond descriptive statistics, logistic regression was performed to examine predictors of bowel necrosis and recurrence. The small sample size necessitated the use of exact logistic regression (ELR) models using Stata’s (version 17) exlogistic function. Confidence intervals were adjusted using the Cornfield method. Due to the rarity of gallstone ileus, a formal power calculation was not feasible. However, statistical techniques appropriate for small sample sizes, such as exact logistic regression and Cornfield-adjusted confidence intervals, were used to reduce Type I and II errors. All analyses were conducted using Stata (Stata Corp, Texas, USA). A two-tailed *p*-value < 0.05 was considered statistically significant.

### 2.7. Ethical Considerations

The study protocol was reviewed and approved by our institutional ethical committee board (approval number: 19377/2025). This study adhered strictly to the ethical principles outlined in the Declaration of Helsinki. Informed consent requirements were waived due to the retrospective nature of this study, ensuring patient anonymity and confidentiality.

## 3. Results

### 3.1. Patient Demographics and Clinical Presentation

This study included 14 patients (age range: 62–84 years; mean age: 73.2 ± 6.5 years; 10 females, 71.4%, and 4 males, 28.6%) diagnosed surgically with gallstone ileus resulting from gallstones impacted within the intestinal lumen. All patients underwent imaging studies comprising plain abdominal radiography, ultrasonography, and high-resolution computed tomography (CT).

### 3.2. Imaging Modalities and Diagnostic Findings Comprising Plain Abdominal Radiography, Ultrasonography, and High-Resolution Computed Tomography

The CT scans identified distinct gallstone calcification patterns: dense (fully calcified) gallstones in six patients (43%), slight opacification in one patient (7%), and rim calcification in seven patients (50%). Rim-calcified stones had the highest correlation with histologically confirmed fibrotic and inflamed tissue along fistulous tracts (RR 1.42, 95% CI: 1.05–1.93, *p* = 0.048). Subgroup analysis revealed that females over 75 had a higher frequency of rim-calcified stones (60%) compared to younger patients or males (*p* = 0.042). Among patients with cholecystoduodenal fistulas, 75% presented within 48 h of symptom onset, suggesting a more acute clinical course. Conversely, jejunal or colonic fistulas were associated with delayed presentation (>72 h) and increased bowel compromise.

### 3.3. Gallstone Calcification Patterns

**Figure 1:**
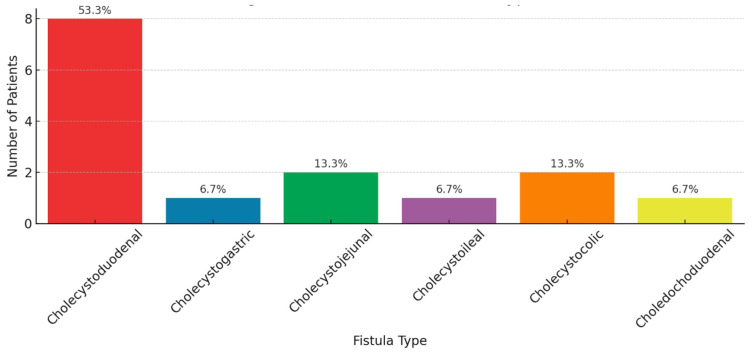
Types of biliary-enteric fistulae identified on CT and confirmed histopathologically. This figure shows the prevalence of different biliary-enteric fistulae. Cholecystoduodenal fistulas were most frequent (57.1%), while others, like cholecystogastric, choledochoduodenal, and cholecystoileal, were less common (7.1% each).

Radiological-pathological analysis of biliary-enteric fistulae showed cholecystoduodenal fistula in eight patients (57.1%), cholecystogastric fistula in one patient (7.1%), cholecystojejunal fistula in two patients (14.3%), cholecystoileal fistula in one patient (7.1%), cholecystocolic fistula in two patients (14.3%), and choledochoduodenal fistula in one patient (7.1%) (Figure 1). Pathological examination confirmed chronic inflammatory changes, epithelial loss, and granulation tissue in all fistula tracts detected on imaging, with a diagnostic concordance rate of 92.8% (13/14 patients). Two patients with recurrence had dense-calcified stones initially and no concurrent cholecystectomy. Logistic regression demonstrated that gallstone size >3.5 cm was a significant predictor of recurrence (OR 2.7; 95% CI: 1.1–6.6; *p* = 0.038), independent of fistula type.

### 3.4. Fistula Type Distribution and Radiological-Pathological Correlation

**Figure 2:**
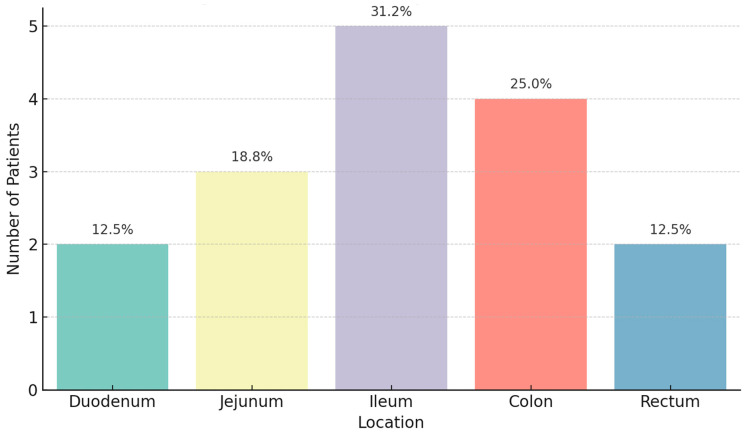
Gallstone impaction locations. The chart highlights where gallstones became lodged. The ileum was the most frequent site (35.7%), followed by the colon (28.6%), jejunum (21.4%), and the duodenum and rectum (14.3% each).

Gallstone impaction sites demonstrated significant variability (*p* = 0.039, Chi-square test), with impact occurring in different sites: in the duodenum (*n* = 2, 14.3%), jejunum (*n* = 3, 21.4%), ileum (*n* = 5, 35.7%), colon (*n* = 4, 28.6%), and rectum (*n* = 2, 14.3%) (Figure 2). Impaction in the ileum was associated with the highest relative risk for bowel wall necrosis confirmed histologically (RR 2.0, 95% CI: 1.1–3.6).

**Figure 3:**
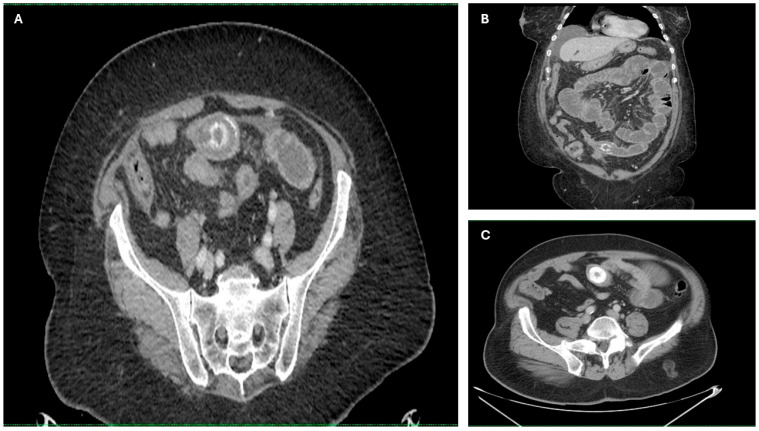
Radiological features of enteric gallstones in biliary ileus. (**A**) Axial CT section demonstrating a highly radiopaque enteric gallstone located within the intestinal lumen, consistent with biliary ileus. (**B**,**C**) Coronal and axial reconstructed CT images further illustrating the presence of an enteric gallstone causing intestinal obstruction—features characteristic of biliary ileus. Acquisition with 5 mm slice thickness and 3 mm reconstruction. Images have been cropped and zoomed for optimal visualization while maintaining the original scale; a 10 mm scale bar is included in each panel.

**Figure 4:**
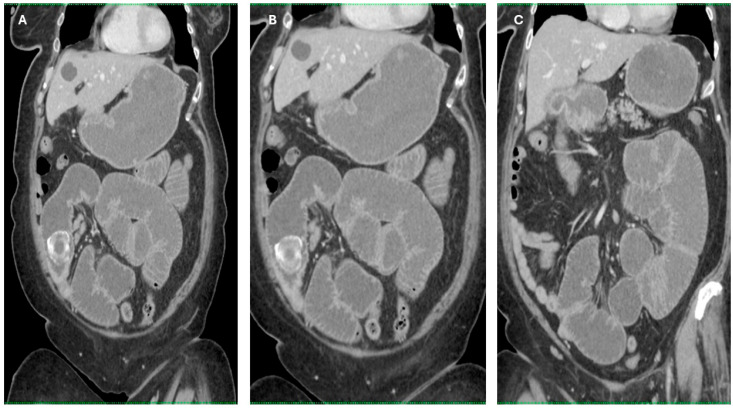
Enteric gallstone associated with cholecystoduodenal fistula. Coronal reconstructed CT images showing an ectopic gallstone within the intestine, secondary to a cholecystoduodenal fistula—a classic radiological finding in biliary ileus. (**A**–**C**) Coronal reconstructions showing multiple distended small bowel loops upstream from the obstruction site, with the intraluminal gallstone visible in the terminal ileum. Acquisition with 5 mm slice thickness and 3 mm reconstruction. Images have been cropped and zoomed for optimal visualization while maintaining the original scale; a 10 mm scale bar is included in each panel.

**Figure 5:**
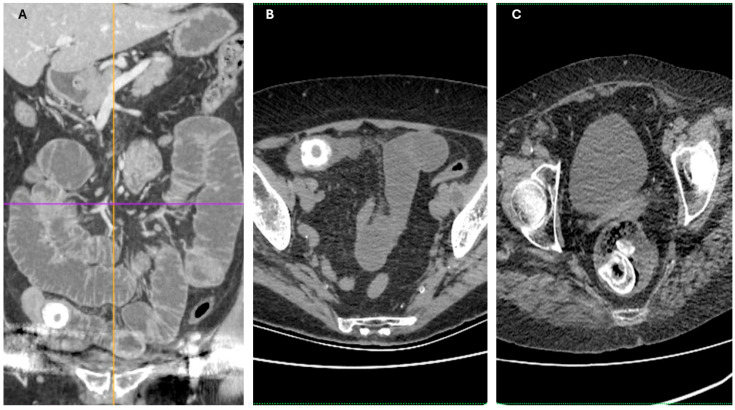
Distal migration of enteric gallstones in biliary ileus. (**A**,**B**) Coronal and axial CT images depicting a highly radiopaque enteric gallstone located in the projection of the colon-biliary ileus. (**C**) Axial CT section showing a radiopaque enteric gallstone in the projection of the rectal ampulla, indicating distal migration—biliary ileus. Acquisition with 5 mm slice thickness and 3 mm reconstruction. Images have been cropped and zoomed for optimal visualization while maintaining the original scale; a 10 mm scale bar is in-cluded in each panel.

Imaging findings were consistent with biliary ileus, characterized by the presence of ectopic gallstones within the gastrointestinal tract. As shown in Figure 3A–C, axial and coronal CT images demonstrated radiopaque enteric gallstones. Figure 4 highlights a gallstone associated with a cholecystoduodenal fistula. Further axial and coronal sections (Figure 5A–C) show additional enteric gallstones, including one in the rectal ampulla.

### 3.5. Site of Gallstone Impaction

Two patients (14.3%) experienced recurrence of gallstone ileus due to subsequent gallstone migration, neither having undergone prior cholecystectomy. All surgically extracted stones measured greater than 3 cm (mean size: 3.6 ± 0.4 cm). Clinical outcomes included successful relief of obstruction in all patients with no perioperative mortality. Follow-up at 6 months showed no new complications in 85.7% of patients.

## 4. Discussion

This study highlights the complementary roles of CT imaging and histopathological analysis in diagnosing and managing gallstone ileus. Our key findings, supported by robust statistical methods, warrant reflection against the established literature.

### 4.1. Clinical Presentation

Gallstone ileus often follows an insidious and intermittent course, manifesting primarily through nonspecific gastrointestinal symptoms. Patients frequently complain of nausea, vomiting, vague abdominal pain, and distension as the stone migrates through the digestive tract [14,15,16]. Rarely, erosion into the gastroduodenal artery may cause upper gastrointestinal bleeding, characterized by hematemesis, coffee-ground emesis, or melena. Bowel perforation and overt peritonitis are exceedingly uncommon at initial presentation.

### 4.2. Role of Plain Radiography

Abdominal radiographs are typically the initial diagnostic tool. Rigler’s triad denotes three distinctive radiographic signs—signs of small bowel obstruction, pneumobilia, and ectopic gallstone—although all three are seen concurrently in only 14–53% of cases [17]. Enhanced detection methods such as Rigler’s tetrad (stone displacement) and pentad (dual air–fluid levels) techniques may improve diagnostic sensitivity.

### 4.3. Ultrasonography in Gallstone Ileus

Ultrasound is useful for identifying gallstones and may reveal Rigler’s triad elements. The so-called “double-arch” sign—two parallel hyperechoic lines indicating mucosal and stone interfaces—is considered pathognomonic [18,19].

However, ultrasound’s sensitivity is limited (~74%) and often compromised by bowel gas interference [14].

### 4.4. CT Imaging and Diagnostic Superiority

Contrast-enhanced CT is regarded as the gold standard diagnostic tool, boasting high sensitivity (93%), specificity (100%), and accuracy (99%) in identifying gallstone ileus, far surpassing plain radiography or ultrasound [20]. CT delineates rim-calcified or total-calcified ectopic gallstones, pneumobilia, and the mechanical obstruction characteristic of Rigler’s triad. It also detects biliary-enteric fistulae—most commonly cholecystoduodenal (32.5–96%)—alongside secondary signs like gallbladder wall thickening, collapse, adhesions, and air-fluid levels [14]. Coronal reconstructions further aid in detecting the often-subtle fistulous tracts.

### 4.5. Assessment of Obstructive Features and Complications via CT

CT enables precise measurement of obstruction-level gallstones (typically ≥ 2.5 cm) and the transition point between dilated and collapsed bowel segments, critical for surgical planning. It also highlights complications such as bowel edema, ischemia, free fluid, pneumatosis, and portal venous gas—prognostic signs of advanced disease [21,22]. Early CT detection correlates strongly with prompt intervention and better outcomes.

### 4.6. MRI and Biliary Tree Visualization

Though less practical in emergency contexts, MRI—including MRCP—can delineate fistulae and detect small gallstones (<3 mm), offering a more comprehensive view of biliary anatomy. However, prolonged scan times and limited acute accessibility make CT the preferred modality in urgent cases [10].

### 4.7. Correlation Between Calcification Patterns and Chronic Pathology

The strong association between rim-calcified gallstones and histopathological evidence of chronic inflammatory fistulae (RR 1.42, 95% CI 1.05–1.93, *p* = 0.048) reinforces CT’s ability to reflect underlying tissue changes. Prior meta-analyses report CT sensitivity at 90–93% and specificity at 100% for gallstone ileus, further validating CT as a reliable tool for detecting these chronic features [23,24,25].

### 4.8. Fistula Detection: Imaging vs Histopathology

We observed a 92.8% concordance between CT-detected fistulae and histopathology-confirmed findings, similar to the literature report of high diagnostic accuracy for detecting Rigler’s triad and biliary-enteric fistulae [26]. This concordance underscores the value of CT not just for stone detection but for informing preoperative planning.

### 4.9. Anatomical Predilection and Ischemic Risk

The terminal ileum was the most common impaction site (35.7%), in line with anatomical susceptibility previously described [10,27]. Our finding of a doubled risk of histologically confirmed bowel necrosis when stones lodge in the ileum (RR 2.0, 95% CI 1.1–3.6) highlights the imperative for early surgical intervention to avert complications.

### 4.10. Recurrence and Definitive Surgical Strategy

Our observed recurrence rate of 14.3% exceeds the 5–9% generally reported following enterolithotomy [3,28,29]. This aligns with existing evidence suggesting that enterolithotomy alone may leave residual fistulae or stones untreated, increasing the risk of recurrence. A higher recurrence underscores the potential benefit of interval or one-stage cholecystectomy in patients with acceptable operative risk, consistent with findings from several systematic reviews and case reports indicating this strategy can limit recurrence at the cost of higher perioperative risk [28,30].

### 4.11. Imaging Features: Rigler’s Triad and Acute Presentation

Our imaging data confirm the utility of contrast-enhanced CT in identifying Rigler’s triad and additional complexities such as bowel ischemia. Literature reports CT accuracy nearing 99%, whereas plain films and ultrasound may detect only parts of the triad, with a sensitivity between 74% and 77% when combined [31,32].

In our context, CT findings guided timely surgical decision-making, especially in cases with rim calcification and suspected necrosis.

### 4.12. Clinical Significance and Recommendations

The diagnostic synergy between CT and histopathology, as demonstrated in our study, aligns with broader findings from large-scale reviews. A meta-analysis [33] and recent case series underscore that CT not only detects Rigler’s triad but also provides predictive data regarding chronicity and complications [9]. Our data bolster the view that rim-calcified stones should prompt evaluation for underlying fistulae, which might be overlooked in conventional assessments.

Moreover, our study echoes other studies’ conclusion that isolated enterolithotomy, while effective short term, often fails to address ongoing inflammatory drivers. Given the recurrence observed in our study, even in a small cohort, we support selective interval cholecystectomy in younger or lower-risk patients, following initial obstruction relief [34].

CT imaging should remain the mainstay of diagnostic, especially when rim calcification is detected.

Enterolithotomy alone is appropriate for elderly or high-risk patients, but patients with rim-calcified stones and fistulae should be strongly considered for definitive biliary surgery to reduce recurrence.

Early detection of ileal impaction with ischemia on CT should trigger urgent surgical decompression.

### 4.13. Strengths and Limitations

This study’s strengths include rigorous radiologic-pathologic correlation and precise statistical analysis, both of which are suitable for small cohort analyses. Nevertheless, its retrospective design and limited sample size reduce its statistical power and generalizability. Despite this, consistency with established high-impact studies adds credibility to our conclusions.

The small sample size (*n* = 14) represents a significant limitation, reducing the generalizability of the findings and limiting the ability to draw firm conclusions applicable to broader clinical populations.

#### 4.13.1. Future Directions

Prospective multicenter studies with larger cohorts are needed to refine operative strategies, especially in balancing morbidity [35] and recurrence risks. Comparative trials examining enterolithotomy with or without definitive biliary procedures in patients stratified by CT and histological risk profiles would further elucidate optimal patient-specific pathways.

In terms of future directions, prospective multicenter trials evaluating combined diagnostic pathways (e.g., CT + serum inflammatory markers) could refine risk stratification. Artificial intelligence-assisted CT interpretation might also reduce interobserver variability in identifying subtle fistulous tracts.

#### 4.13.2. Limitations

This study has several limitations. First, the retrospective design inherently introduces selection and information biases, limiting the control over variables and the completeness of data. Second, the relatively small sample size (*n* = 14) reduces statistical power, particularly for subgroup analyses and multivariate modeling, and limits the generalizability of the findings. While statistical adjustments were applied to address small event rates, the risk of Type II error cannot be excluded.

Third, the study was conducted in a single tertiary care center, which may limit external validity due to potential institutional biases in imaging protocols, surgical decision-making, and postoperative care. Fourth, the absence of long-term follow-up data beyond 6 months prevents assessment of late complications or recurrence in the broader clinical course.

Additionally, although imaging-pathology correlation was strong, interobserver variability in radiological interpretation and histopathological grading was not formally assessed, which may introduce subjective bias.

Despite these limitations, the study provides valuable insights into the diagnostic performance of CT and the pathological correlations of gallstone ileus, aligning with findings from high-impact multicenter studies.

## 5. Conclusions

This study underscores the significance of detailed imaging and pathological analysis in diagnosing gallstone ileus. Recognizing distinct gallstone calcification patterns and accurately identifying fistula types and impaction locations assists radiologists and pathologists in predicting clinical outcomes and tailoring surgical strategies. The occurrence of recurrent gallstone ileus suggests the potential value of definitive surgical intervention, such as cholecystectomy, in selected patients to prevent recurrence.

## Data Availability

The datasets analyzed during the current study are available from the corresponding author on reasonable request.

## Abbreviations

CT	Computed Tomography
ELT	Enterolythotomy
MRCP	Magnetic resonance cholangiopancreatography
MRI	Magnetic resonance imaging
SBO	Small bowel obstruction
Stata	Data analysis and statistical software
US	Ultrasound
